# Mutational spectrum in breast cancer associated ***BRCA1*** and ***BRCA2*** genes in Colombia

**Published:** 2017-06-30

**Authors:** Ignacio Briceño-Balcázar, Alberto Gómez-Gutiérrez, Natalia Andrea Díaz-Dussán, María Claudia Noguera-Santamaría, Diego Díaz-Rincón, María Consuelo Casas-Gómez

**Affiliations:** 1 Escuela de Medicina, Universidad de la Sabana, Chía, Colombia; 2 Instituto de Genetica Humana. Facultad de Medicina, Pontificia Universidad Javeriana, Bogotá, Colombia.; 3 Instituto de Referencia Andino, Bogotá, Colombia.

**Keywords:** Breast Neoplasms, genes, BRCA1, BRCA2, DNA Sequence Analysis, Female, DNA Mutational Analysis, Humans, Colombia

## Abstract

**Introduction:**

The risk of developing breast and ovarian cancer is higher in families that carry mutations in BRCA1 or BRCA2 genes, and timely mutation detection is critical.

**Objective:**

To identify the presence of mutations in the Colombian population and evaluate two testing strategies.

**Methods:**

From a total universe of 853 individual blood samples referred for BRCA1 and BRCA2 typing, 256 cases were analyzed by complete direct sequencing of both genes in Myriad Genetics, and the remaining 597 cases were studied by partial sequencing based on founder mutations in a PCR test designed by ourselves ("Profile Colombia").

**Results:**

We found 107 patients carrying deleterious mutations in this group of patients, 69 (64.5%) located in BRCA1, and 38 (35.5%) in BRCA2. Overall, we detected 39 previously unreported mutations in Colombia (22 in BRCA1 and 17 in BRCA2) and only 4 out of the 6 previously reported founder mutations. Sixty four out of 597 patients (10.7%) studied by "Profile Colombia" showed mutations in BRCA1 or BRCA2, and 41/256 patients (16%) showed mutations by complete BRCA1-BRCA2 sequencing.

**Conclusions:**

The spectrum of 44 different mutations in Colombia as detected in our study is broader than the one previously reported for this country. "Profile Colombia" is a useful screening test to establish both founder and new mutations (detection rate of 10.7%) in cases with family history of breast cancer. Complete sequencing shows a detection rate of 16.0%, and should complement the study of the genetic basis of this disease.

## Resumen

**Introducción:**

El riesgo de desarrollar cáncer de mama y cáncer de ovario puede transmitirse en familias que porten mutaciones en los genes BRCA1 o BRCA2. La detección de estas mutaciones permite tomar decisiones oportunas en el ámbito de la medicina preventiva.

**Objetivo:**

Estudiar el espectro de mutaciones en la población colombiana y evaluar dos estrategias de detección.

**Metodos:**

Se incluyeron en total 853 pacientes con diagnóstico de cáncer de mama y con solicitud de análisis de los genes BRCA1 y BRCA2. Un total de 256 pruebas se analizaron mediante secuencia directa completa de estos genes en Myriad Genetics, y las restantes 597 se estudiaron mediante secuencia parcial basada en mutaciones fundadoras a través de la prueba "Perfil Colombia", implementada por nosotros.

**Resultados:**

Se detectaron 107 pacientes portadores de mutaciones en pacientes colombianos, 69 de las cuales estaban localizadas en BRCA1 y 38 en BRCA2. De estas 39 mutaciones son nuevas (22 en BRCA1 y 17 en BRCA2) y solo se hallaron 4 de las 6 mutaciones reportadas previamente como fundadoras en Colombia. En 64/597 pacientes analizados mediante el "Perfil Colombia" se detectaron mutaciones en BRCA1 o BRCA2, así como en 41/256 pacientes que solicitaron la secuenciación completa de los genes BRCA1 y BRCA2.

**Conclusiones:**

El espectro de mutaciones fundadoras en Colombia es más amplio que el reportado anteriormente para este país. El "Perfil Colombia" es una prueba que revela a la vez mutaciones fundadoras y mutaciones nuevas, con una tasa de detección del 10.7%. La secuenciación completa presenta una tasa de detección del 16.0% y puede complementar el diagnóstico de la base genética de esta enfermedad.

## Introduction

Breast cancer is the second most common cancer in the world and the most common type of cancer among women, with an incidence of 1.67 million cases diagnosed in 2012 (25% of all cancers) ^1^. In North America and Western Europe, 1 in 8 or 9 women will develop breast cancer during their lifetime ^2^. In Colombia, cancer incidence and mortality rate in men and women is a growing public health issue ^3^. Nearly 70,890 annual cases of cancer were reported between 2000 and 2006, 54.41% of which were diagnosed in women, the main locations being breast, cervix, thyroid, stomach and colon ^3^ ^,^ ^4^. According to Rodríguez *et al*., the age-standardized incidence of ovarian cancer in Colombia is estimated as 10.1 cases per 100,000 individuals per year, and approximately 11.5% of all ovarian cancer patients carry a single BRCA1 mutation known to be a Colombian founder mutation ^5^ ^-^ ^8^. Previous studies have registered incidence rates of 5-10% of hereditary cases in women under 45 years ^9^ ^,^ ^10^. 

According to results obtained by Torres *et al*., BRCA1 and BRCA2 gene mutations have been found affecting between 5 and 8% of breast cancer Colombian patients, and 10% of patients with ovarian cancer ^6^ ^-^ ^8^. However, women with deleterious mutations in any of these genes are at 87% risk of developing breast cancer and 68% risk of ovarian cancer ^8^. In addition to this, BRCA1 and BRCA2 mutations increase the risk of lymphomas, prostate, larynx, pancreas, gastrointestinal tract and liver cancer ^2^ ^,^ ^11^ ^-^ ^14^.

The normal function of BRCA1 and BRCA2 genes is DNA repair, transcription and recombination, all of which prevent cancer development as part of the tumor suppressor group of genes. BRCA1 protein helps prevent the cells from growing in an uncontrolled way by repairing nuclear DNA with proteins encoded by RAD51 and BARD1, and is associated with RNA polymerases in their C-terminal domain allowing its interaction with histone deacetylase complexes participating in cell proliferation, developmental processes and transcription regulation. BRCA2 protein also controls cell growing, and interacts with RAD51 in DNA repair and homologous recombination ^10^.

In some cases genetic mutations in BRCA1 and BRCA2 are inherited, which may generate uncontrolled proliferation of a single cell and contribute to the development of cancer^7^. The development of malignancy (carcinogenesis) is a multi-step process, characterized by a progression of genetic alterations in a single cell line, were cells respond inappropriately to normal regulatory mechanisms ^7^ ^,^ ^15^.

BRCA1 gene, located in chromosome 17 (locus: 17q12-q21), was identified in 1990 after studying 23 families with a total of 143 cases of breast and ovarian cancer ^16^, while BRCA2 gene was identified in 1994 in chromosome 13 locus:13q12-q13 through a study that analyzed 15 families at high risk of familial breast cancer, including male cases ^17^ ^-^ ^19^.

Given the high relation of these mutations with the development of breast cancer, their incidence in Colombia has been studied for many years. In 2007, six deleterious founder mutations were reported, 50% of them where present in families with multiple cases of breast cancer and 33% in families who had breast cancer and/or ovarian cancer, for a total of 53 families included in the study ^8^. The three most frequent deletions or transitions were 3450delCAAG and A1708E in BRCA1, and 3034delACAA in BRCA2 ^8^. 

In a second report conducted in 780 patients with sporadic breast cancer (no criteria for family breast cancer), the six founder mutations previously described were studied and it was found that mutations A1708E and 3450delCAAG in BRCA1 were positive in 2.9% and mutation 3034delACAA in BRCA2 was positive in 1.3% of the patients ^20^. Penetrance at 50 years of age was calculated up to 19% for BRCA1 and 10% for BRCA2. In a recent review in Latin America, Ossa and Torres ^6^ reported a restricted spectrum of founder mutations in different countries, and a specific spectrum of the above cited 3 founder mutations in Colombia (A1708E and 3450delCAAG in BRCA1 and 3034delACAA in BRCA2).

An unpublished diagnostic PCR test called "Profile Colombia" was developed by our group as a screening strategy in Colombia by sequencing upstream and downstream framing fragments around the reported founder mutations ^8^. This strategy allowed the low cost detection of patients at high risk of developing cancer, and revealed the presence of four of the six most common mutations in Colombia (2 for BRCA1 and 2 for BRCA2) as described in Torres *et al*., as well as new mutations in the current population in this country. In mutation carrier patients, prevention strategies can be implemented to lower the risk by up to 98% ^8^ ^,^ ^20^.

The majority of previous studies have been performed in the population of Bogotá, but Hernández *et al*., developed an investigation involving nearly 244 patients with breast cancer from Medellín, where only three mutations, two in BRCA1 -one with family history and one sporadic- and one in BRCA2, a sporadic case, were found in 1.2% of the patients. Of the three mutations reported in Medellín, the first two were included in "Profile Colombia" and the third, 5844del5, had not been previously reported in individuals with Colombian descent ^10^.

The main objective of this study was to determine the mutational spectrum in BRCA1 and BRCA2 genes in Colombia, and compare two diagnostic strategies to detect patients at high risk of developing cancer and establish prevention programs. 

## Materials and Methods

### Patient Population

Eight hundred and fifty three female patients with breast cancer diagnosed at any age in a 6 year period -between 2009 and 2014- were selected for this study from different regions of Colombia, with the following criterium: All samples sent to a reference laboratory in Colombia for analysis of mutations in the *BRCA1* and *BRCA2* genes, including requests for "Perfil Colombia" and requests for "Complete sequence of the *BRCA1* and *BRCA2* genes". Applications from laboratories outside Colombia (mainly Ecuador and Panama) were excluded. This protocol was performed by convenience as a descriptive analytical study.

Participating centers approved the analytical protocol with the endorsement of the research committee of Instituto de Referencia Andino (IRA), and every patient who participated in the study gave written informed consent. After signing the informed consent, a blood sample was obtained from each patient, and referenced to IRA in Bogotá.

### Extraction, amplification and DNA sequencing

In a global universe of 853 individual blood samples referred for BRCA1 and BRCA2 typing, DNA extraction was performed from all blood samples. Of these, 256 cases (30%) were analyzed by complete direct sequencing of both genes in Myriad Genetics^®^ Laboratories, and the remaining 597 cases (70%) were studied by partial sequencing based on founder mutations in a test designed by IRA Laboratories in Bogotá ("Profile Colombia"). The second procedure was carried out based on primers designed along selected BRCA1 and BRCA2 sequences in order to include the six most common mutations in Colombia (3450delCAAG, A1708E, 3034delACAA, 6076delGTTA, 6503delTT, W31X) as reported by Torres *et al.*, as well as upstream and downstream sequences in short (100-120 bp) framing fragments around the reported founder mutations. The partial sequences obtained were analyzed in a 4.8 Sequencher^®^ program. BRCA1 and BRCA2 complete sequencing was analyzed by Myriad Genetics^®^. The analytical report and results were both referred to patients with a recommendation for a genetic counseling session. 

### Data analysis

Results of molecular analysis of the BRCA1 and BRCA2 gene sequences were registered in Excel^®^ tables and mutation frequencies were subsequently defined. This protocol was performed by convenience as a descriptive analytical study.

## Results

BRCA1 and BRCA2 genetic analyses from 853 patients were performed, of which 256 (30.0%) were analyzed by total direct sequence test and the remaining 597 (70.0%) were studied by partial sequence-based on founder mutations in the PCR analysis called "Profile Colombia". This study detected 107 patients carrying mutations of which 69 (64.5%) were located in BRCA1 and 38 (35.5%) in BRCA2. Additionally, among patients analyzed with "Profile Colombia", 209 (35.0%) showed a G5337A polymorphism in BRCA1 and 54 (9.0%) patients a A3199G polymorphism in BRCA2, the latter registered as a "class 1" mutation in NIH-BIC database. Overall, 39 new mutations were detected (22 in BRCA1 and 17 in BRCA2) which had not been reported in the previous studies of founder mutations in Colombia ^6^ ^,^ ^8^ ^,^ ^20^ in 2007, 2009 and 2016 (Table 1). Sixty four out of 597 patients showed different BRCA1 or BRCA2 deleterious mutations (10.7%) by "Profile Colombia", and 41 out of 256 patients (16.0%) showed different deleterious mutations by complete sequencing of the BRCA1 and BRCA2 genes.

**Table 1:** Mutations detected in BRCA1 and BRCA2 in Colombia and their clinical relevance according to NCBI (NIH-BIC and ClinVar). Pathogenic mutations as reported in international databases appear in bold.

	Patients	%	Mutation registry (BIC)	Mutation registry (ClinVar)
BRCA1*				
3450 delCAAG**	13	18.8	Class 5	Pathogenic
A1708E**	27	39.1	Pending	Pathogenic
G3031A	1	1.4	NR	NR
T3014C	1	1.4	NR	NR
C5214T	1	1.4	Pending	Pathogenic
1163 delTG	1	1.4	NR	NR
C5141T	3	4.3	NR	NR
1793 delA	4	5.8	Class 5	Pathogenic
5221 delTG	1	1.4	Class 5	Pathogenic
5221 delT	1	1.4	NR	NR
5637 delG	1	1.4	NR	NR
C39R (234 T>C)	2	2.9	Pending	NR
W1508X (4642 G>A)	1	1.4	Class 5	Pathogenic
5154 delTTTTC	1	1.4	Class 5	NR
E720X (2277G>T)	1	1.4	Class 5	NR
N1742S	1	1.4	NR	Uncertain
2881 delGACA	1	1.4	NR (report: 2883 delACAG)	NR
1499 insA	1	1.4	Class 5	Pathogenic
V1145F	2	2.9	NR	NR
2031 delG	1	1.4	Class 5	Pathogenic
K168E	1	1.4	NR	NR
5356 delT	1	1.4	NR	NR
R1835X (5622 C>T)	1	1.4	Pending	Pathogenic
W1712X (5255G>A)	1	1.4	Class 5	Pathogenic
Total	69	63.9		
BRCA2***				
3034 delACAA**	8	21.1	Class 5	Pathogenic
6076 delGTTA**	1	2.6	Class 5	Pathogenic
6503 delTT**	0	0.0	Class 5	Pathogenic
W31X**	0	0.0	Pending	NR
T289A	1	2.6	NR	NR
C6448A	1	2.6	Pending	Benign
C3046T	1	2.6	Class 5	Pathogenic
V572L	1	2.6	NR	Uncertain
P218L	1	2.6	NR	NR
C6328T	3	7.9	Pending	Benign
T10K	1	2.6	NR	Uncertain
2929 delC	1	2.6	NR	Benign
3154 TC>AT	1	2.6	Pending	NR
C5972T	11	28.9	Class 1	Benign
T1011R (3260C>G)	1	2.6	Pending	Conflicting interpretations
4772 delA	1	2.6	NR	NR
6310 delGA	1	2.6	NR (report: 6310 delGAAGA)	Benign
A5996C	1	2.6	Pending	Conflicting interpretations
6062 insG	1	2.6	NR	NR
S1630X (5117C>G)	1	2.6	Class 5	NR
N570S (1937A>G)	1	2.6	NR	Uncertain
Totasl	38	35.5		

* 22 new mutations in Colombia

** Profile Colombia

*** 17 new mutations in Colombia

**Figure 1:**
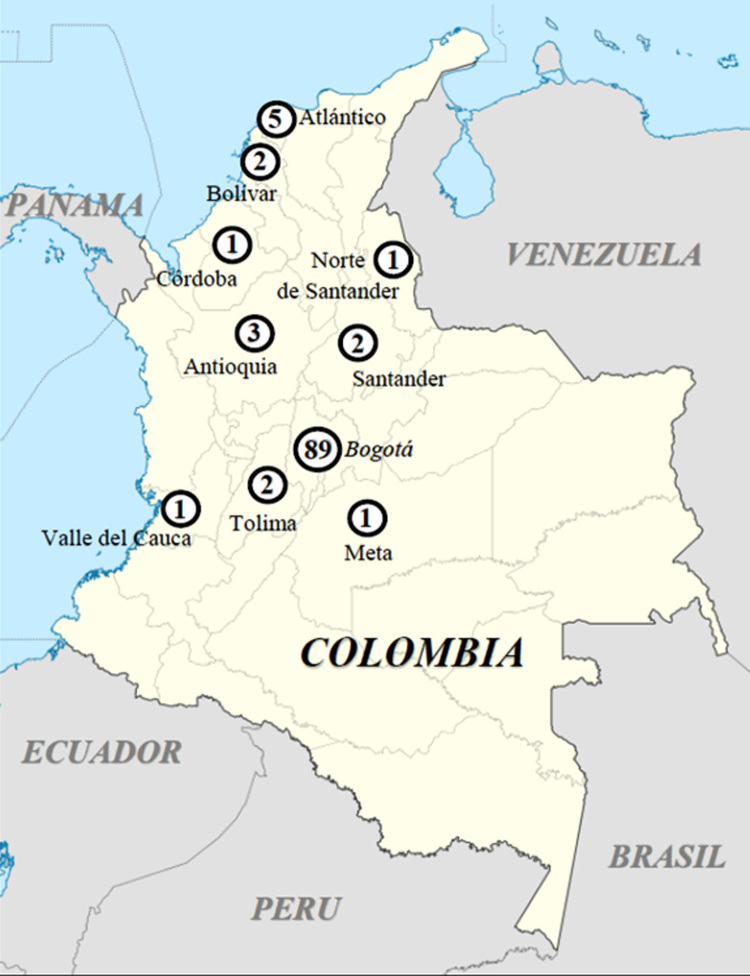
Mutation distribution in Colombia. Numbers of patients carrying mutations in each region are specified inside the dark circles.

Most patients with BRCA1 and BRCA2 mutations among 107 positive individuals came from Bogotá (89/263 -33.8%- patients, corresponding to 10.43% of the global number of 853 patients), followed by Barranquilla and Atlántico (5/18 -27.8%- patients, i. e. 0.59% positive in the global sample), Medellín in Antioquia (3/8 -37.5%- patients, i. e. 0.35% of the global sample), Bucaramanga in Santander (2/8 -25%- patients, i. e. 0.23% of the Colombian sample), Cartagena in Bolívar (2/7 -28.6%- patients, i. e. 0.23% of the sample), Ibagué in Tolima (2/5 -40%- patients, i. e. 0.23% of the sample), Cúcuta in North of Santander (1/1 patient, i. e. 0.12% of the sample), Cali in Valle del Cauca (1/11 -27.3%- patient, i. e. 0.12% of the sample), Villavicencio in Meta (1/3 -33.3%- patient, i. e. 0.12% of the sample) and Montería in Córdoba (1/2 -50%- patient, i. e. 0.12% of this sample). Two patients were received from Pereira in Risaralda, who showed no specific BRCA1/BRCA2 mutations. Figure 1 represents the spectrum of the distribution of mutations found in the different departments of origin in Colombia, and Table 2 specifies the particular mutations found in each region.

**Table 2:** Mutations detected in BRCA1 and BRCA2 in different departments of Colombia.

Department of origin in Colombia	BRCA1	Patients	BRCA2	Patients	Total	% mutation carriers in Colombian sample
Bogotá (D. C.)	3450delCAAG	13	3034delACAA	7	89	10.43
	A1708E	23	C5972T	7		
	1793delA	4	C6328T	3		
	5221delTG	1	A1937G	1		
	G3031A	1	6076delGTTA	1		
	1163delTG	1	T289A	1		
	C5141T	1	C6448A	1		
	5637delG	1	C3045T	1		
	5154delTTTTC	1	P218L	1		
	E720X	1	T10K	1		
	N1742S	1	2929delC	1		
	2881delGACA	1	V572L	1		
	1499insA	1	C3260G	1		
	R1835X	1	6310delGA	1		
	2031delG	1	4772delA	1		
	K168E	1	A5996C	1		
	T234C	2	C5117G	1		
	V1145F	2	6062insG	1		
Antioquia	C5141T	1	C5972T	1	3	0.35
	W1508X	1				
Atlántico	A1708E	1	3154TC>AT	1	5	0.59
	C5141T	1	C5972T	1		
	W1712X	1				
Bolívar	A1708E	1			2	0.23
	T3014C	1				
Córdoba	5356delT	1			1	0.12
Meta			3034delACAA	1	1	0.12
Norte de Santander			C5972T	1	1	0.12
Santander	A1708E	2			2	0.23
Tolima	C5214T	1			2	0.23
	5221delT	1				
Valle del Cauca			C5972T	1	1	0.12
Total number of patients		69		38	107	12.5%

Among 107 affected patients, 69 (64.5%) were found to carry mutations in BRCA1, of which 40 (58.0%) were either 3450delCAAG or A1708E, previously reported as founder mutations ^6^ ^,^ ^8^ ^,^ ^20^, and 38 (35.5%) in BRCA2, of which 9 mutations (23.7%) correspond to 3034delACAA or else 6076delGTTA, both previously defined as founder mutations and included in "Profile Colombia". 

The global deleterious mutational spectrum included 24 different locations in BRCA1 and 22 in BRCA2, of which 22 are new for BRCA1 and 17 are new for BRCA2 in Colombia (Table 2), as related to preliminary reports of founder mutations in Colombian populations ^6^ ^,^ ^8^ ^,^ ^20^. Furthermore, within non-founder mutations, a high prevalence was observed for the G5337A (BRCA1) and A3199G (BRCA2) polymorphisms, with 209 and 54 cases each on 35.0% and 9.0% of the patients respectively. 

We identified the presence of a simultaneous deleterious mutation and a polymorphism in BRCA1 in the same patient and of simultaneous polymorphisms in both BRCA1 and BRCA2; simultaneous gene mutations and polymorphisms in the same patient were not observed in BRCA2. Both G5337A and A3199G polymorphisms present in BRCA1 and BRCA2 genes respectively, have not yet been reported as deleterious mutations (i.e. mutations directly involved in the development of breast cancer), and in consequence they should be considered as benign polymorphisms.

## Discussion

The present study estimated the prevalence of BRCA1 and BRCA2 gene mutations in a broad Colombian population of 853 breast cancer patients. For some patients (30.0%), complete sequencing of BRCA1 and BRCA2 genes, and for the rest (70.0%) an extended profile of six previously reported funder mutations were performed in order to identify eventual immediate upstream and downstream gene mutations previously unreported in Colombia.

We identified 43 deleterious mutations in BRCA1 and BRCA2 (39 new and 4 previously reported founder mutations), all of them involved in the development of breast cancer. Among these, 24 were identified in BRCA1, in addition to the G5337A polymorphism, which, given its high prevalence, requires further study to determine its clinical implication. On the other hand, 19 mutations were reported in BRCA2, in addition to the A3199G polymorphism which was also found in a high proportion in this population.

Although the clinicians that referred the patients would not report specific clinical criteria taken into account as the basis for their request of genetic testing in a total of 853 patients included in this study, more than a third of the sample (39.3%) had a mutation or polymorphism and, among these, 49 cases (5.7%) showed founder mutations reported by the 2007 study ^8^, which are part of "Profile Colombia".

The most frequent mutations in BRCA1 were A1708E (27 patients) and 3450delCAAG (13 patients), followed by four patients with 1793delA, C5141T (3 patients), C39R (234T>C) and V1145F (2 patients each). As for BRCA2, the most frequent mutations besides the previously reported founder mutation 3034delACAA (8 patients), were C5972T (11 patients) and 6328C>T (3 patients), all of which could be considered founder mutations in Colombia (Tables 1 and ^2^). 

Although the largest proportion of scientific literature suggests that when cancer is highly related to genetic factors and is attributable to specific founder mutations in a given population, it is possible to perform an inexpensive screening -as compared to the cost of the complete sequencing of each gene ^15^, the results obtained in this study show that 84.3% of the patients carrying a mutation in BRCA1 or BRCA2 did not show any one of the previously defined founder mutations. This fact may be the consequence of an admixed population as the one that inhabits Colombia. We therefore suggest complete sequencing of BRCA1 and BRCA2 genes in patients with negative results in the screening test denominated "Profile Colombia", where up to 10.7% of the population would be detected by this particular PCR analysis as carriers of deleterious mutations as showed in our study, in order to clear an important subset of genetic risk factors.

Although there were limitations associated with the pre-analytical phase of this study, as data from clinical records were scarce and no follow-up was undertaken directly by us in patients who tested positive for a mutation associated in the development of breast cancer, and even if the genetic sequencing of restricted fragments of BRCA1 and BRCA2 will necessarily leave out an indeterminate proportion of possible mutations in these genes, the record of 39 new deleterious mutations by two complementary molecular testing protocols, which have not been previously reported in Colombia, suggests that Colombians should not be considered as restricted as other populations such as the Finnish or the Ashkenazi Jews, as could have been deduced from the previous report of a small number of founder mutations ^8^. However, it is clear that there is a high prevalence of mutations that can be associated to founder events, namely: 3450delCAAG (13 patients in our study) and A1708E (27 patients in our study) in BRCA1, and 3034delACAA (8 patients in our study) and C5972T (11 patients in our study) in BRCA2 ^6^. As shown in Table 1, the clinical significance of each mutation was investigated in the NCBI databases (BIC and ClinVar), and at least 12/24 (50.0%) BRCA1 mutations and 4/19 (21.0%) BRCA2 mutations were found to be registered as "pathogenic", the others being either uncertain, conflicting or not yet reported in these databases. Only 5 mutations in BRCA2, namely C6448A, C6328T, 2929delC, C5972T and 6319delGA were found to be reported as "benign" in one of the databases, and only in one case (C5972T), both BIC and ClinVar define this mutation as "benign" and "class 1". 

Finally, the large proportion of patients clinically diagnosed with breast cancer in whom no mutations were found, especially among those with complete sequence of the BRCA1 and BRCA2 genes, supports the fact that the development of breast and ovarian cancer not only depends on a reduced spectrum of genetic factors associated with mutations in these particular genes, and that other genetic and non-genetic factors must be considered. 

## Conclusion

Due to the worldwide high breast cancer incidence and the diversity of mutations identified in a Colombian population, early detection of carriers by profiling and subsequent gene sequencing should allow for monitoring and prevention strategies long before the development of disease. "Profile Colombia" (which includes upstream and downstream neighbor sequencing of previously identified founder mutations) has proved to be a low cost screening PCR analysis useful for local detection of supplementary deleterious and founder mutations. 
