# Supplementary material for: Mutational spectrum in breast cancer associated BRCA1 and BRCA2 genes in Colombia
Source: Colomb Med (Cali). 2017 Jun 30;48(2):58–63. (PMC5625557)
Supplement: Translation

# Translation: Espectro de mutaciones en los genes ***BRCA1*** y ***BRCA2*** asociados a cáncer de mama en Colombia

## Introducción

El cáncer de mama es el segundo cáncer más común en el mundo y el más común entre las mujeres, con una incidencia de 1.7 millones de casos diagnosticados en el 2012 (25% de todos los cánceres) ^1^.

En América del Norte y en Europa Occidental, 1 de 8-9 mujeres desarrollarán cáncer de mama durante su vida ^2^. En Colombia, la tasa de incidencia de mortalidad de cáncer en hombres y mujeres es un problema de salud pública ^3^. Alrededor de 70,890 casos de cáncer anuales fueron reportados entre el 2000-2016, 54.41% de los cuales fueron diagnosticados en mujeres, siendo sus localizaciones principales mama, cérvix, tiroides, estómago y cólon ^3^^,^^4^. De acuerdo con Rodríguez *et al*, la edad estándar de incidencia de carcinoma de ovario en Colombia se estimó como 10.1 casos por cada 100,000 individuos por año, y aproximadamente 11.5% de todos los carcinomas de ovario se hallaron en pacientes que portan una sola mutación de BRCA1, conocida como una mutación fundadora en Colombia ^5^^,^^8^. Estudios previos registraron tasas de incidencia de 5-10% de casos hereditarios en mujeres por debajo de los 45 años de edad ^9^^,^^10^. 

De acuerdo con los resultados obtenidos por Torres *et al*, se han encontrado mutaciones en los genes BRCA1 y BRCA2 afectando entre el 5 y 8% de los pacientes con cáncer de mama en Colombia, y en 10% de los pacientes con carcinoma de ovario ^6^^,^^8^. Sin embargo, mujeres con mutaciones deletéreas en cualquiera de estos genes tienen 87% de riesgo de desarrollar carcinoma de mama y 68% de riesgo de desarrollar carcinoma de ovario ^8^. Adicionalmente, mutaciones del BRCA1 y BRCA2 incrementan el riesgo de linfoma, carcinoma de próstata, de laringe, de páncreas, de tracto gastrointestinal y carcinoma de hígado ^2^^,^^11^^-^^14^.

Las funciones normales de los genes BRCA1 y BRCA2 son la reparación, transcripción y recombinación del ADN, las cuales previenen el desarrollo del carcinoma como parte del grupo de genes supresores de tumores. La proteína BRCA1 ayuda a prevenir el crecimiento incontrolado de las células reparando el ADN nuclear con proteínas codificadas por los genes RAD51 y BARD1, y está asociada con las polimerasas ARN en el dominio C-terminal, permitiendo su interacción con complejos de histona deacetilasas, participando en la proliferación celular, procesos de desarrollo y regulación de transcripción. La proteína BRCA2 también controla el crecimiento celular e interactúa con el RAD51 en reparación del ADN y recombinación homóloga ^10^.

En algunos casos, mutaciones genéticas en BRCA1 y BRCA2 son heredadas, lo cual podría generar proliferación descontrolada de una sola célula y contribuir al desarrollo del cáncer ^7^. El desarrollo de la malignidad (carcinogénesis) es un proceso multifacético, caracterizado por la progresión de alteraciones genéticas en una sola línea celular, donde las células responden inapropiadamente a un mecanismo regulatorio normal ^7^^,^^15^. 

El gen BRCA1, localizado en el cromosoma 17 (locus: 17q12-q21), fue identificado en 1990 después de estudiar 23 familias con un total de 143 casos de carcinoma de mama y ovario ^16^, mientras que el gen BRCA2 fue identificado en 1994 en el cromosoma 13 (locus: 13q12-q13), a través de un estudio que analizó 15 familias con un elevado riesgo para cáncer de mama, incluyendo casos masculinos ^17^^-^^19^.

Dada la relación de estas mutaciones con el cáncer de mama, su incidencia en Colombia ha sido estudiada durante muchos años. En 2007 se reportaron seis mutaciones deletéreas fundadoras, 50% de ellas presentes en familias con múltiples casos de cáncer de mama y 33% en familias que tenían cáncer de mama y/o de ovario, para un total de 53 familias incluidas en el estudio ^8^. Tres de las más frecuentes deleciones o transiciones fueron: 3450delCAAG y A1708E en BRCA1 y 3034delACAA en BRCA2 ^8^.

En un segundo reporte conducido en 780 pacientes con cáncer de mama esporádico (sin criterio para cáncer de mama familiar), las seis mutaciones fundadoras previamente descritas fueron estudiadas, y se encontró que las mutaciones A1708E y 3450delCAAG en BRCA1 fueron positivas en el 2.9% de los casos, y la mutación 3034delACAA en BRCA2 fue positiva en 1.3% de los pacientes ^20^. La penetrancia a los 50 años de edad fue calculada hasta el 19% para BRCA1 y 10% para BRCA2. En estudios recientes en Latinoamérica, Ossa y Torres^6^ reportaron un espectro restringido de mutaciones fundadoras en diferentes países, y un espectro específico de las tres mutaciones fundadoras nombradas previamente en Colombia (A1708E y 3450delCCAG en BRCA1 y 3034delACAA en BRCA2).

Una prueba de PCR diagnóstica original denominada "Perfil Colombia" fue desarrollada por nuestro grupo como una estrategia de tamizaje en Colombia secuenciando corriente arriba y corriente bajo los fragmentos enmarcados alrededor de las mutaciones fundadoras reportadas previamente ^8^. Esta estrategia permitió la detección a bajo costo de pacientes con alto riesgo de desarrollar cáncer, y reveló la presencia de 4 de las 6 mutaciones más comunes en Colombia (dos para BRCA1 y dos para BRCA2) de acuerdo con el reporte de Torres *et al*., además de encontrar nuevas mutaciones en la población actual en este país. En pacientes portadores de mutación, la detección temprana puede ser implementada para disminuir el riesgo hasta un 98% ^8^^,^^20^.

La mayoría de los estudios previos fueron realizados en la ciudad de Bogotá, pero Hernández *et al.*, desarrollaron una investigación que involucró alrededor de 244 pacientes con cáncer de mama de Medellín, donde solo tres mutaciones, dos en BRCA1 -una con historia familiar y una esporádica- y una en BRCA2, caso esporádico, fueron encontrados en el 1.2% de los pacientes. De las tres mutaciones reportadas en Medellín, las primeras dos fueron incluidas en el "Perfil Colombia" y la tercera, 5844del5, no se había reportado previamente en individuos de ascendencia colombiana ^10^.

El objetivo principal de este estudio fue determinar el espectro mutacional en los genes BRCA1 y BRCA2 en Colombia, y evaluar dos estrategias diagnosticas para proponer una metodología de detección de pacientes de alto riesgo de desarrollo de cáncer y establecer programas de prevención.

## Materiales y Métodos

### Población de estudio

Ochocientos cincuenta y tres pacientes del género femenino con cáncer de mama diagnosticados a cualquier edad en un periodo de seis años (entre 2009-2014) fueron seleccionadas para este estudio de diferentes regiones de Colombia bajo el siguiente criterio: Todas las muestras enviadas a un laboratorio de referencia en Colombia para análisis de mutaciones en los genes BRCA1 y BRCA2, incluyendo órdenes para "Perfil Colombia" y órdenes para "Secuenciación completa de genes BRCA1 y BRCA2", excluyendo aplicaciones de laboratorios fuera de Colombia, principalmente de Ecuador y Panamá. Este protocolo fue realizado por conveniencia, como un estudio analítico descriptivo.

Los centros participantes aprobaron el protocolo analítico con respaldo del comité del Instituto de Referencia Andino (IRA), y cada paciente participante diligenció el consentimiento informado por escrito. Después de firmar el consentimiento informado, se obtuvo una muestra de sangre de cada paciente y esta fue referenciada al IRA en Bogotá.

### Extracción, amplificación y secuenciación de ADN

En un universo global de 853 muestras de sangre individuales referidas para tipificación de BRCA1 y BRCA2se realizó la extracción de ADN de todas las muestras. De estas, 256 casos (30%) fueron analizados por secuenciación directa para ambos genes en Laboratorios Myriad Genetics^®^ (USA), los 597 casos restantes (70%) fueron estudiados por secuenciación parcial basada en las mutaciones fundadoras reportadas previamente en la población, en una prueba diseñada por laboratorios IRA en Bogotá ("Perfil Colombia"). El segundo procedimiento fue llevado a cabo con primers diseñados sobre secuencias seleccionadas de BRCA1 y BRCA2 para incluir las seis mutaciones más comunes en Colombia (3450delCAAG, A1708E, 3034delACAA, 6076delGTTA, 6503delTT, W31X), como fue reportado por Torres *et al.* Adicionalmente, se realizaron secuencias cortas (100-120 pares de bases) en el marco de los fragmentos alrededor de las mutaciones fundadoras mencionadas. Las secuencias parciales obtenidas fueron analizadas en un el programa Sequencher^®^ 4.8. La secuenciación completa para BRCA1 y BRCA2 fue llevada a cabo por Myriad Genetics^®^. El reporte analítico y sus resultados fueron entregados a los pacientes con recomendación de una sesión de consejería genética. 

### Análisis de datos

Los resultados de los análisis moleculares de los genes BRCA1 y BRCA2 fueron registrados en tablas Excel^®^ y las frecuencias de mutación fueron subsecuentemente definidas. Este protocolo fue realizado por conveniencia en el marco de un estudio descriptivo analítico

## Resultados

Se realizaron análisis genéticos en los genes BRCA1 y BRCA2 de 853 pacientes colombianos, de los cuales 256 (30.0%), fueron analizados por secuenciación directa total y los 507 restantes (70.0%) fueron estudiados por secuenciación parcial basada en las mutaciones fundadoras en el análisis de PCR denominado "Perfil Colombia". Este estudio detectó 107 pacientes portando mutaciones en estos genes, de las cuales 69 (64.5%) fueron localizadas en BRCA1, y 38 (35.5%) en BRCA2. Adicionalmente, entre los pacientes analizados con "Perfil Colombia", 209 (35.0%) mostraron el polimorfismo G5337A en BRCA1, y 54 (9.0%) de los pacientes el polimorfismo A3199G en BRCA2, este último registrado como mutación "clase 1" en la base de datos del NIH-BIC. En total fueron detectadas 39 nuevas mutaciones (22 en BRCA1 y 17 en BRCA2), las cuales no habían sido reportadas en estudios previos de mutaciones fundadoras en Colombia ^6^^,^^8^^,^^20^ en los años 2007, 2009 y 2016 (Tabla 1). Sesenta y cuatro de 597 pacientes (i. e. 10,7%) mostraron diferentes mutaciones deletéreas de BRCA1 o BRCA2 por "Perfil Colombia", y 41 de los 256 pacientes (16%), mostraron diferentes mutaciones deletéreas por secuenciación completa de los genes BRCA1 y BRCA2 en Myriad Genetics^®^.

**Tabla 1:** Mutaciones detectadas en BRCA1 y BRCA2 en Colombia y su relevancia clínica de acuerdo con el NCBI (NIH-BIC y ClinVar). Las mutaciones patogénicas se reportan tal y como están reportadas en bases de datos internacionales, y aparecen en negrilla.

	Pacientes	%	Registro mutacional (BIC)	Registro mutacional (ClinVar)
BRCA1*				
3450 delCAAG**	13	18.8	Clase 5	Patogénica
A1708E**	27	39.1	Pendiente	Patogénica
G3031A	1	1.4	NR	NR
T3014C	1	1.4	NR	NR
C5214T	1	1.4	Pendiente	Patogénica
1163 delTG	1	1.4	NR	NR
C5141T	3	4.3	NR	NR
1793 delA	4	5.8	Clase 5	Patogénica
5221 delTG	1	1.4	Clase 5	Patogénica
5221 delT	1	1.4	NR	NR
5637 delG	1	1.4	NR	NR
C39R (234 T>C)	2	29	Pendiente	NR
W1508X (4642 G>A)	1	1.4	Clase 5	Patogénica
5154 delTTTTC	1	1.4	Clase 5	NR
E720X (2277G>T)	1	1.4	Clase 5	NR
N1742S	1	1.4	NR	Uncertain
2881 delGACA	1	1.4	NR (reporte: 2883 delACAG)	NR
1499 insA	1	1.4	Clase 5	Patogénica
V1145F	2	2.9	NR	NR
2031 delG	1	1.4	Clase 5	Patogénica
K168E	1	1.4	NR	NR
5356 delT	1	1.4	NR	NR
R1835X (5622 C>T)	1	1.4	Pendiente	Patogénica
W1712X (5255G>A)	1	1.4	Clase 5	Patogénica
TOTAL	69	63.9		
BRCA2***				
3034 delACAA**	8	21.1	Clase 5	Patogénica
6076 delGTTA**	1	2.6	Clase 5	Patogénica
6503 delTT**	0	0.0	Clase 5	Patogénica
W31X**	0	0.0	Pendiente	NR
T289A	1	2.6	NR	NR
C6448A	1	2.6	Pendiente	Benigna
C3046T	1	26	Clase 5	Patogénica
V572L	1	2.6	NR	Incierta
P218L	1	2.6	NR	NR
C6328T	3	7.9	Pendiente	Benigna
T10K	1	2.6	NR	Incierta
2929 delC	1	2.6	NR	Benigna
3154 TC>AT	1	2.6	Pendiente	NR
C5972T	11	289	Class 1	Benigna
T1011R (3260C>G)	1	2.6	Pendiente	en conflicto
4772 delA	1	2.6	NR	NR
6310 delGA	1	2.6	NR (reporte: 6310 delGAAGA)	Benign
A5996C	1	2.6	Pendiente	En conflicto
6062 insG	1	2.6	NR	NR
S1630X (5117C>G)	1	2.6	Clase 5	NR
N570S (1937A>G)	1	26	NR	Incierta
Total	38	35.5		

La mayoría de los pacientes con mutaciones BRCA1 y BRCA2 (107 individuos positivos) enviaron su muestra desde Bogotá (89/263 -33.8%- correspondientes al 10.4% del número global de 853 pacientes), seguidos por quienes la enviaron desde Barranquilla y Atlántico (5/18 -27.8%-, es decir 0.59% de pacientes positivos en la muestra global), desde Medellín en Antioquia (3/8 -37.5%- pacientes, es decir 0.35% de la muestra global), desde Bucaramanga en Santander (2/8 -25%- pacientes, es decir 0.23% de la muestra colombiana), desde Cartagena en Bolívar (2/7 -28.6%- pacientes, es decir, 0.23% de la muestra), desde Ibagué en Tolima (2/5 -40%- pacientes, es decir 0.23% de la muestra), desde Cúcuta en Norte de Santander (1/1 pacientes, es decir, 0.12% de la muestra), desde Cali en Valle del Cauca (1/11 -27,3%- pacientes, es decir, 0.12% de la muestra), desde Villavicencio en Meta (1/3 -33.3%- pacientes, es decir 0.12% de la muestra y desde Montería en Córdoba (1/2 -50.0%- pacientes, es decir 0.12 de esta muestra). Dos pacientes fueron recibidos de Pereira en Risaralda, y no mostraron mutaciones específicas en BRCA1/BRCA2. La Figura 1 representa el espectro de distribución en las mutaciones encontradas en diferentes departamentos de origen en Colombia. La Tabla 2 especifica las mutaciones particulares encontradas en cada región. Entre los 107 pacientes afectados, 69 (64.5%) portaron mutaciones en BRCA1, de las cuales 40 (58.0%) fueron 3450delCAAG o A1708E, previamente reportadas como mutaciones fundadoras ^6^^,^^8^^,^^20^, y 38 (35.5%) portaban mutaciones en BRCA2. Entre todas estas, 22 fueron nuevas para BRCA1 y 17 fueron nuevas para BRCA2 en Colombia (Tabla 2), de acuerdo con en el reporte preliminar de mutaciones fundadoras en poblaciones colombianas ^6^^,^^8^^,^^20^. Además, entre las mutaciones no fundadoras, se halló una alta prevalencia para polimorfismos G5337A (BRCA1) y A3199G (BRCA2), con 209 y 54 casos cada uno, en 25.0% y 9.0% de los pacientes respectivamente.

**Figura 1:** Distribución de mutaciones BRCA1 y BRCA2 en Colombia. El número de pacientes portadores de mutaciones en cada región se halla insertado en los circulos negros.

**Tabla 2:** Mutaciones detectadas en BRCA1 y BRCA2 en diferentes departamentos de Colombia.

Departamento de origen en Colombia	BRCA1	Pacientes	BRCA2	Pacientes	Total	% portadores de mutación en la muestra de Colombia
Bogotá (D. C.)	3450delCAAG	13	3034delACAA	7	89	10.43
	A1708E	23	C5972T	7		
	1793delA	4	C6328T	3		
	5221delTG	1	A1937G	1		
	G3031A	1	6076delGTTA	1		
	1163delTG	1	T289A	1		
	C5141T	1	C6448A	1		
	5637delG	1	C3045T	1		
	5154delTTTTC	1	P218L	1		
	E720X	1	T10K	1		
	N1742S	1	2929delC	1		
	2881delGACA	1	V572L	1		
	1499insA	1	C3260G	1		
	R1835X	1	6310delGA	1		
	2031delG	1	4772delA	1		
	K168E	1	A5996C	1		
	T234C	2	C5117G	1		
	V1145F	2	6062insG	1		
Antioquia	C5141T	1	C5972T	1	3	0.35
	W1508X	1				
Atlántico	A1708E	1	3154TC>AT	1	5	0.59
	C5141T	1	C5972T	1		
	W1712X	1				
Bolívar	A1708E	1			2	0.23
	T3014C	1				
Córdoba	5356delT	1			1	0.12
Meta			3034delACAA	1	1	0.12
Norte de Santander			C5972T	1	1	0.12
Santander	A1708E	2			2	0.23
Tolima	C5214T	1			2	0.23
	5221delT	1				
Valle del Cauca			C5972T	1	1	0.12
Número total de pacientes		69		38	107	12.5%

Identificamos también la presencia simultánea de mutaciones deletéreas y polimorfismos en el mismo paciente en BRCA1, y, por otra parte, de polimorfismos simultáneos en ambos genes, BRCA1 y BRCA2; no se observaron mutaciones y polimorfismos simultáneos en el mismo paciente en BRCA2. Estos polimorfismos, G5337A y A3199G, presentes en genes BRCA1 y BRCA2 respectivamente, aún no han sido reportados como mutaciones deletéreas (es decir, mutaciones directamente involucradas en el desarrollo de cáncer de mama), y en consecuencia deben ser considerados por lo pronto como polimorfismos benignos.

## Discusión

El presente estudio estimó la prevalencia de mutaciones en los genes BRCA1 y BRCA2 en una vasta población colombiana de 853 pacientes con cáncer de mama. Para algunos pacientes (30%), se procedió con la secuenciación completa de genes BRCA1 y BRCA2, y para el resto (70%), se realizó un perfil extendido ("Perfil Colombia") de 6 de las mutaciones fundadoras previamente reportadas en orden de identificar mutaciones inmediatas corriente arriba y corriente abajo, no reportadas previamente en Colombia.

Identificamos en total 43 mutaciones deletéreas en BRCA1 y BRCA2 (39 nuevas además de las 4 mutaciones fundadoras previamente reportadas), eventualmente involucradas en el desarrollo de cáncer de mama. Sobre estas, 24 fueron identificadas en BRCA1, en adición al polimorfismo G5337A, el cual, dada su alta prevalencia, requiere estudios adicionales para determinar su implicación clínica. Por otro lado, se reportaron 19 mutaciones en BRCA2, en adición al polimorfismo 3199G, el cual también fue hallado en una alta proporción de la población. 

Aunque los médicos que refirieron los pacientes no reportan los criterios clínicos que los llevaron a solicitar los análisis genéticos más allá del cáncer de mama en sus pacientes, en un total de 853 individuos incluidos en el estudio, más de un tercio de la muestra (39.3%) presentó una mutación o polimorfismo y, entre estos, 49 casos (5.7%) presentaron mutaciones fundadoras reportadas por el estudio de 2007 ^8^, las cuales hacen parte del "Perfil Colombia".

Las mutaciones más frecuentes en BRCA1 fueron A1708E (27 pacientes) y 3450delCAAG (13 pacientes), seguidas de cuatro pacientes con 1793delA, 3 pacientes con C5141T y 2 pacientes para C39R (234T>C) y para V1145F. En cuanto al BRCA2, las mutaciones más frecuentes además de la mutación fundadora 3034delACAA en 8 pacientes, se encontraron: C5972T (11 pacientes) y 6328C>T (3 pacientes). Todas estas podrían ser consideradas mutaciones fundadoras en Colombia en razón al número de pacientes portadoras (Tablas 1 y ^2^).

Aunque la mayor proporción de literatura científica sugiere que el cáncer está altamente relacionado con factores genéticos y es atribuible a mutaciones fundadoras específicas en una población dada, es posible desarrollar un tamizaje de bajo costo, en comparación a la secuenciación completa de cada gen ^15^; los resultados obtenidos en este estudio muestran que el 84.3 % de los pacientes portadores de mutaciones en BRCA1 y BRCA2 no presentaron ninguna mutación fundadora de las previamente descritas. Este hecho podría ser la consecuencia de una población mezclada como los habitantes de Colombia. Por esta razón sugerimos la secuenciación completa de los genes BRCA1 y BRCA2 en pacientes con resultados negativos del tamizaje denominado "Perfil Colombia", a través del cual se podría detectar hasta el 10.7% de la población portadora y en riesgo a través de este análisis restringido de PCR, como se describe en nuestro estudio. De esta manera se podría descartar un subgrupo importante de importantes factores genéticos de riesgo.

Aunque hubo limitaciones asociadas con la fase pre-analítica de este estudio, ya que la información de las historias clínicas fue escasa y no hubo seguimiento realizado directamente por nosotros a los pacientes que obtuvieron resultados positivos para una mutación asociada con el desarrollo de cáncer, e incluso si la secuenciación genética de fragmentos restringidos de BRCA1 y BRCA2 necesariamente deja por fuera una proporción indeterminada de posibles mutaciones en estos genes, el registro de 39 nuevas mutaciones deletéreas identificadas a través de dos protocolos complementarios de análisis molecular, las cuales no habían sido previamente reportadas en Colombia, sugiere que la población colombiana no debería ser considerada tan restringida genéticamente como otras poblaciones como los finlandeses o los judíos ashkenazi como podría haberse deducido de los reportes previos de un pequeño número de mutaciones fundadoras para esta población de la esquina norte de Suramérica ^8^. Sin embargo, es claro que hay una alta prevalencia de mutaciones que pueden estar asociadas a eventos fundadores conocidos, tales como: 3450delCAAG (13 pacientes en nuestro estudio) y A1708E (27 pacientes en nuestro estudio) en BRCA1, y 3034delACAA (8 pacientes en nuestro estudio) y C5972T (11 pacientes en nuestro estudio) en BRCA2 ^6^. Como se ilustra en la Tabla 1, la significancia clínica de cada mutación fue investigada en las bases de datos del NBCI (BIC y ClinVar), y al menos 12/24 (50%) mutaciones de BRCA1 y 4/19 (21%) mutaciones de BRCA2 fueron registradas como "patogénicas". Las otras se clasificaron como "inciertas", "en conflicto" o aun no reportadas en estas bases de datos. Solo cinco mutaciones en BRCA2, conocidas (C6448A, C6328T, 2929delC, C5972T, 6319delGA) fueron encontradas y reportadas como benignas en una de las bases de datos y en un solo caso (C5972T), ambas bases, BIC y ClinVar, definen esta mutación como "benigna" y también de "clase 1".

Finalmente, la mayoría de los pacientes diagnosticados clínicamente con cáncer de mama en quienes no se hallaron mutaciones, específicamente entre los analizados con secuenciación completa de genes BRCA1 y BRCA2, soportan el hecho de que el desarrollo de cáncer de mama y de ovario, no solo depende en un espectro reducido de factores genéticos asociados con mutaciones en estos genes en particular, y que otros factores genéticos y no genéticos deben ser considerados en la evaluación diagnóstica de cada población.

## Conclusión

Debido a la alta incidencia de cáncer de mama a nivel mundial, y a la alta diversidad de mutaciones identificadas en una población colombiana, la detección temprana de portadores realizada por secuenciación de genes y la aplicación de un perfil genético determinado (como el "Perfil Colombia") deben permitir estrategias de prevención y monitoreo mucho antes del desarrollo de esta enfermedad. El "Perfil Colombia" (que incluye la secuenciación vecina inmediata, corriente arriba y corriente abajo, de las mutaciones fundadoras previamente identificadas), ha probado ser un análisis de PCR útil como tamizaje, y de bajo costo, para la detección local de mutaciones deletéreas y fundadoras conocidas y suplementarias.
